# Survival outcomes of patients with diffuse large B-cell lymphoma undergoing autologous stem cell transplantation in Germany: real-world evidence from an administrative database between 2010 and 2019

**DOI:** 10.3389/fonc.2024.1432310

**Published:** 2024-10-09

**Authors:** Jan-Michel Heger, Peter Borchmann, Sybille Riou, Barbara Werner, Michael S. Papadimitrious, Jörg Mahlich

**Affiliations:** ^1^ Department I of Internal Medicine, Center for Integrated Oncology Aachen Bonn Cologne Duesseldorf, University of Cologne, Medical Faculty and University Hospital Cologne, Cologne, Germany; ^2^ Health Economics and Outcomes Research, Miltenyi Biomedicine, Bergisch Gladbach, Germany; ^3^ Health Economics and Health Services Research, Team Gesundheit Gesellschaft für Gesunheitsmanagement mbH, Essen, Germany; ^4^ Duesseldorf Institute for Competition Economics (DICE), Heinrich-Heine-University Düsseldorf, Düsseldorf, Germany

**Keywords:** diffuse large B-cell lymphoma, autologous stem cell transplantation, survival, real-world evidence, claims data, Germany

## Abstract

**Background:**

Limited real-world evidence is available for patients with diffuse large B-cell lymphoma (DLBCL) who received an autologous stem cell transplantation (ASCT) in Germany.

**Objectives:**

This study aims to describe the real-world survival outcomes of patients with DLBCL who received ASCT in Germany after diagnosis.

**Design:**

This study is a retrospective database analysis covering the period between 2010 and 2019.

**Methods:**

Unadjusted overall survival (OS) was plotted using the Kaplan–Meier estimator for the overall population and stratified by relapse status. A Cox regression was run to identify factors that influence OS.

**Results:**

A total of 112 patients received an ASCT, with the average time from first-line treatment to ASCT being 11.7 months. The median OS estimated by Kaplan–Meier was 83.4 months for the entire cohort. The only variable that significantly reduced the OS was the presence of subsequent treatment after ASCT in a time-dependent model.

**Conclusion:**

OS after ASCT for DLBCL patients in Germany is higher than previously reported and may still be considered a valid option for carefully selected patients with relapsed/refractory DLBCL.

## Introduction

Autologous stem cell transplantation (ASCT) following high-dose chemotherapy can still be a treatment option for a well-defined group of patients with diffuse large B-cell lymphoma who relapse or are refractory after front-line therapy, even in the era of chimeric antigen receptor (CAR)-T-cell therapies. Both lisocabtagene maraleucel and axicabtagene ciloleucel, which were approved by the EMA in 2022 and 2023 for this indication (1), have demonstrated superiority over the standard of care (2, 3).

Up to 50% of patients will experience a relapse after ASCT, with the majority occurring within the first year (4). Despite the high number of relapses after ASCT, a relapse 1 year post-ASCT is associated with better survival outcomes. Limited real-world evidence relating to ASCT is available for Germany, with the exception of a single-center study in aggressive B-cell lymphomas, including diffuse large B-cell lymphoma (DLBCL). This study was conducted at the University Hospital Muenster between 2002 and 2019 (5). It reported a mean overall survival (OS) of 55 months for DLBCL patients who received ASCT. While the Kaplan–Meier (KM) curves indicated an OS benefit for patients who relapsed late (defined as ≥ 12 months after ASCT), this difference was not statistically significant. As single-center studies might suffer from limited generalizability, our study utilized a large health insurance claims database covering 6.7 million people to examine survival in German DLBCL patients after ASCT.

## Materials and methods

This is a secondary analysis of a health economics study that is described elsewhere (6, 7). Briefly, the database used for this analysis is a subset of the German Statutory Health Insurance (SHI) population, covering 6.7 million people between 2010 and 2019. It was used for health service research covering multiple indications (8, 9). Deaths are reported in this database, which is not the case for claims databases in many other countries. We identified patients diagnosed with DLBCL who received an ASCT (index date) after front-line treatment. Unadjusted OS from ASCT was plotted using the KM estimator for both the overall population and stratified by (1) the presence of subsequent treatment line (yes/no) and (2) time from the start of first-line therapy to ASCT (< 12 months/≥ 12 months). Patients with a subsequent treatment line were labeled as nondurable responders, while those without a subsequent treatment line were identified as potential responders. The KM estimator was also stratified by early (subsequent treatment line is initiated < 12 months after ASCT) and late relapse (≥ 12 months after ASCT) for the nondurable responder subgroup. A Cox regression with the following covariates—age, gender, Charlson Comorbidity Index (CCI), time from the start of first-line therapy to ASCT, and presence of subsequent treatment after ASCT—was performed to identify factors influencing OS. Model fit was assessed using several diagnostic parameters, such as Akaike’s information criterion (AIC), the Wald test, or the supremum test, to examine whether the proportional hazard assumption holds. If the assumption was violated, a model with time-dependent variables was applied (10). This is an extension of the Cox model and provides a feasible alternative to a landmark analysis, where subjects would have been excluded from the analysis (11). This approach avoids both the problem of selecting a landmark time and misclassification errors (12).

## Results

### Descriptive statistics

Among the 124 patients who received a hematopoietic stem cell transplantation (HSCT) after an initial diagnosis of DLBCL, 112 underwent ASCT and 12 received allogeneic HSCT (which is not included in this study). For patients who had one previous treatment, the average (median) follow-up time after the index was 32.0 (26.0) months. The majority (60.7%) of the cohort were men, and the mean age of patients at the index was 55.2 years old (range: 18–74). The mean pre-index CCI score was 7.8 (range: 2–16). A total of 39.3% (*N* = 44) received at least one subsequent treatment after ASCT, while 60.7% (*n* = 68) did not. The majority (65.9%) of patients who received subsequent treatments were men. The mean age of this subgroup was 56.0 years old, and the mean CCI score was 8.0. For the cohort without subsequent treatment, 57.4% were men, the mean age was 54.6 years old, and the mean CCI score was 7.7. The average time from first treatment to ASCT was 11.7 months for the entire cohort, and 11.0 and 12.1 months for the subgroups with and without subsequent treatment, respectively. The average time to subsequent treatment after ASCT was 13.5 months. In 72.7% of the cases (*N* = 32), subsequent treatment was initiated within 1 year after ASCT (early relapse), while 27.3% (*N* = 12) had subsequent treatment initiated more than 1 year after ASCT.

### Survival analysis

**Figure 1:**
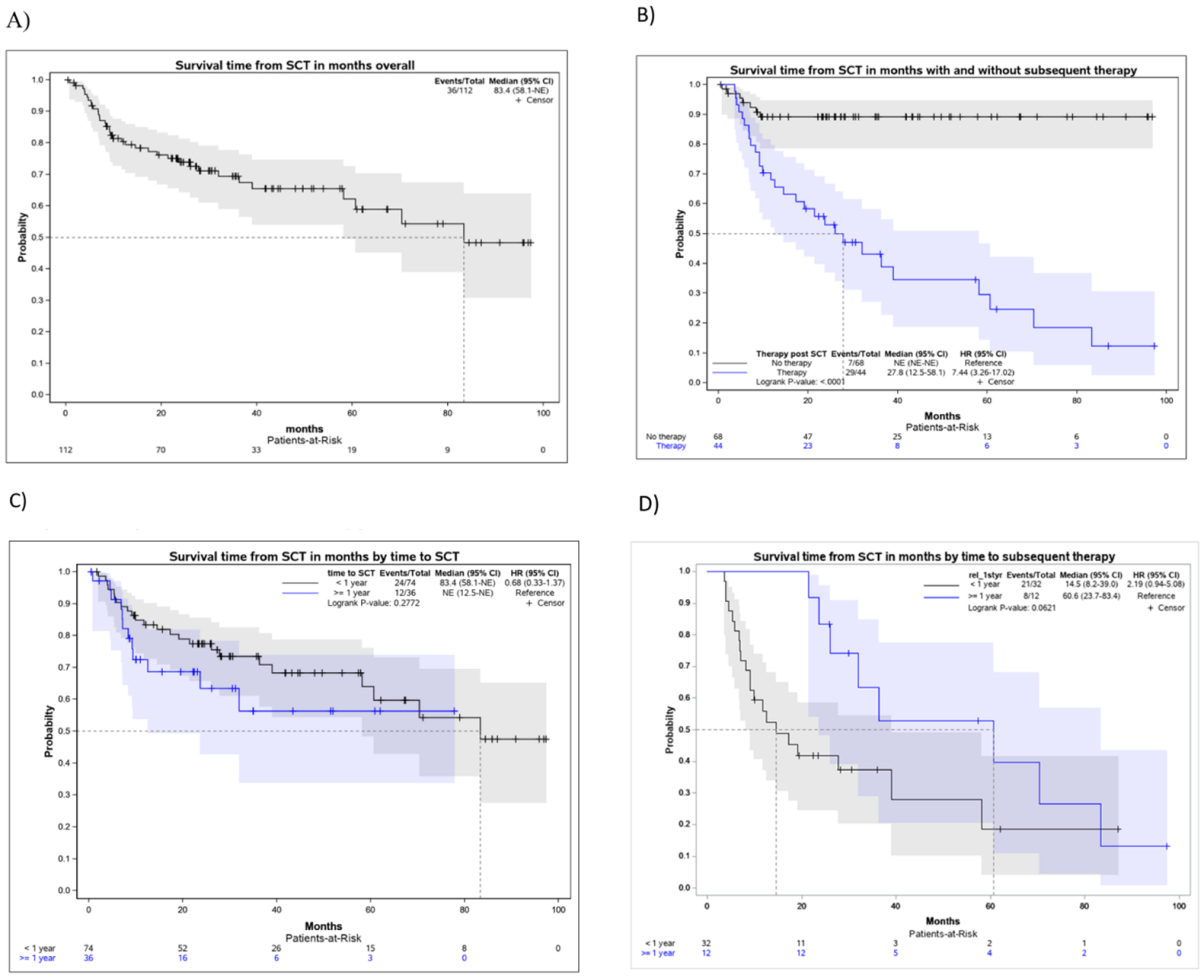
Kaplan–Meier curves for the survival times in months after ASCT. **(A)** Kaplan–Meier curve for the survival time in months from the first ASCT, overall cohort. **(B)** Kaplan–Meier curve for the survival time in months from first ASCT, stratified by subsequent therapy after ASCT. **(C)** Kaplan–Meier curves for the survival times in months from first ASCT onward, stratified by early/late relapse after first-line therapy. **(D)** Kaplan–Meier curves for the survival times in months from the first ASCT onward, stratified by early/late relapse after ASCT.

The median OS was 83.4 months, with a 5-year survival rate of 62.2%, as estimated by the KM curve (**Figure 1A**). The median OS for the group with subsequent treatment was 27.8 months, and it was not estimated for patients without additional treatments (*p* < 0.0001). The KM curve for patients without subsequent treatment reached a plateau of just under 90% (**Figure 1B**). Stratification by the time of relapse after front-line treatment shows a slightly better (but insignificant) hazard ratio (HR) compared to those with early relapse (< 12 months). However, the median for late relapses was not reached, and survival times cannot be compared (**Figure 1C**). Turning to the subgroup of patients who had a relapse after ASCT, the median OS, as estimated by the KM curve, were 14.5 and 60.6 months for early and late relapse, respectively; however, these were not significantly different at the 5% level (*p* = 0.0621) (**Figure 1D**).

Factors influencing OS were studied using a Cox regression. Several models were examined for the entire cohort and stratified by relapse status, using the following variables: age, gender, CCI, time to receive ASCT in months, and time to subsequent treatment in months (if applicable). The best fit was observed for a time-dependent model with “subsequent treatment” as a time-dependent covariate. The model was run for the entire cohort, and **Table 1** reports the results. The only significant variable influencing OS was “subsequent treatment” (*p* < 0.001), with a hazard ratio of 24.24, i.e., the risk of dying at any time point is 24.24 times higher among those who have already received subsequent therapy compared to those who have not, holding all else equal.

**Table 1:** Cox regression with a time-dependent covariate (*n* = 112).

Variables	*p*-value	Hazard ratio (HR)	95% HR confidence interval
Subsequent treatment after ASCT (time-dependent)	< 0.0001	24.236	10.092–58.204
Gender (men vs. women)	0.7465	1.127	0.545–2.333
Age (years)	0.2436	1.026	0.983–1.072
CCI	0.695	0.973	0.847–1.117
Time from initial diagnosis to ASCT (months)	0.1045	1.001	1.000–1.002

AIC: 300.2 (without covariates) and 235.8 (with covariates). Wald test: 51.983 (Chi-square), < 0.0001 (p-value).

ASCT, autologous stem cell transplantation; CCI, Charlson Comorbidity Index; AIC, Akaike’s information criterion.

## Discussion

To our knowledge, this is the first large-scale database study on the survival of DLBCL patients who underwent ASCT in Germany. Our findings indicate that patients in Germany are treated according to recommended treatment guidelines, as ACST is applied as second-line treatment. The 5-year survival rate in this study was 62.2%, and OS after ASCT was 83.4 months, which is broadly in line with the literature. For the USA, OS after ASCT was 64.8 months (5.4 years) for patients treated at the Mayo Clinic (4) or 72 months, as reported by Eldjerou et al., drawing on data from the CIBMTR registry (13). A study from Japan that included elderly patients (median age: 64) reported a 3-year survival rate of 49.6%, compared to 69.4% in our study (14). A more recent Japanese study covered a slightly younger patient cohort and utilized data from the Japan Society for Hematopoietic and Cellular Therapy Registry. The authors reported 5-year survival rates between 57% and 65%, depending on the high-dose chemotherapy regimen [ ranimustine, etoposide, cytarabine, melphalan (MEAM), ranimustine, carboplatin, etoposide, cyclophosphamide (MCEC), or cyclophosphamide, etoposide, melphalan, dexamethasone (LEED)] (15). A recent study from Belgium presented slightly higher 5-year survival rates of 66%–69% (16).

On the other hand, the only study conducted in Germany observed lower values for both the 5-year survival rate (50%) and mean OS (55 months) (5). One potential explanation for the differences might be the higher mean age in the population studied by Wullenkord et al. (5) (63 years old) compared to our study (55 years old). In addition, their study was conducted in a single tertiary teaching hospital, whereas our study accessed a healthcare claims database for all of Germany. Furthermore, high-risk patients are typically referred to tertiary teaching hospitals, and they may not be considered representative of German clinical practice.

We observed a 10% mortality rate in the 10 months following ASCT, possibly as a result of neutropenia (5). This 10% mortality estimate is higher than expected but still within the range of what had been reported in previous research (17). Mortality was zero approximately 1 year after receiving ASCT for those without subsequent treatment. Furthermore, we observed that 39.3% of patients received subsequent treatment after ASCT in our analysis, compared to 28% in Wullenkord et al. (5).

Turning to prognostic factors, the stratified survival analysis suggests that a late relapse after ASCT is associated with an OS advantage of 46.1 months compared to early relapse, which supports previous findings (3). However, due to the small size of this subpopulation, this difference is significant only at the 10% level and not at the conventional 5% level. In Wullenkord et al. (5), the KM curves indicate an OS benefit of more than 5 years for patients who relapsed late after ASCT. However, they also determined that the OS benefit was not statistically significant. Recall that, due to data availability, we used the time from DLBCL treatment to ASCT, which is different from the time to relapse to ASCT used as eligibility criteria and for high-risk classification in the CAR-T trials. OS did not significantly differ between patients who relapsed early or late after first-line therapy. As the median OS for patients with a late relapse (after first-line therapy) has not yet been reached after more than 6 years, it is possible that these patients may demonstrate a long-term survival benefit. The results of the Cox regression confirm a strong association between relapse status after ASCT and OS, demonstrating that relapsed patients have a poor prognosis. No other significant relationships were found.

This retrospective study has several limitations. Claims data, in general, are not designed for research purposes and may suffer from coding errors (18). They also include only a very limited set of medical parameters, and the cause of death is not recorded. For instance, Eastern Cooperative Oncology Group (ECOG) performance status and the amount of infused cells have been reported to influence survival (4, 19) but were not included in our Cox regression due to data limitations. Other potential success factors of ASCT, such as chemosensitivity (20), were not captured in our data. Finally, and probably most importantly, the study covers the time before the advent of new treatment options such as tafasitamab (21), glofitamab (22), or loncastuximab tesirine (23) and other promising therapies (24). Even more relevant treatments are CAR-T-cell therapies (lisocabtagene maraleucel and axicabtagene ciloleucel), which are effective treatments for relapsed or refractory patients who relapse within 12 months of the end of first-line therapy (25). While there may be an urgent medical need for DLBCL patients who cannot receive ASCT, our results, echoing Shadman et al. (1), suggest that CAR-T-cell therapy and ASCT can coexist in that ASCT can still be a valid option for patients presenting with a late relapse, showing a 5-year survival rate of around 55%.

## Data Availability

Database is commercially available but not for free. Requests to access these datasets should be directed to barbara.werner@teamgesundheit.de.
